# Effects of H_2_ and N_2_ treatment for B_2_H_6_ dosing process on TiN surfaces during atomic layer deposition: an *ab initio* study†

**DOI:** 10.1039/c8ra02622j

**Published:** 2018-06-08

**Authors:** Hwanyeol Park, Sungwoo Lee, Ho Jun Kim, Daekwang Woo, Se Jun Park, Kangsoo Kim, Euijoon Yoon, Gun-Do Lee

**Affiliations:** Department of Materials Science and Engineering, Seoul National University Seoul 08826 Korea eyoon@snu.ac.kr gdlee@snu.ac.kr; Department of Mechanical Engineering, Dong-A University Busan 49315 South Korea; Memory Thin Film Technology Team, Giheung Hwaseong Complex, Samsung Electronics 445-701 South Korea; Research Institute of Advanced Materials and Inter-University Semiconductor Research Center, Seoul National University Seoul 08826 South Korea

## Abstract

For the development of the future ultrahigh-scale integrated memory devices, a uniform tungsten (W) gate deposition process with good conformal film is essential for improving the conductivity of the W gate, resulting in the enhancement of device performance. As the memory devices are further scaled down, uniform W deposition becomes more difficult because of the experimental limitations of the sub-nanometer scale deposition even with atomic layer deposition (ALD) W processes. Even though it is known that the B_2_H_6_ dosing process plays a key role in the deposition of the ALD W layer with low resistivity and in the removal of residual fluorine (F) atoms, the roles of H_2_ and N_2_ treatments used in the ALD W process have not yet been reported. To understand the detailed ALD W process, we have investigated the effects of H_2_ and N_2_ treatment on TiN surfaces for the B_2_H_6_ dosing process using first-principles density functional theory (DFT) calculations. In our DFT calculated results, H_2_ treatment on the TiN surfaces causes the surfaces to become H-covered TiN surfaces, which results in lowering the reactivity of the B_2_H_6_ precursor since the overall reactions of the B_2_H_6_ on the H-covered TiN surfaces are energetically less favorable than the TiN surfaces. As a result, an effect of the H_2_ treatment is to decrease the reactivity of the B_2_H_6_ molecule on the TiN surface. However, N_2_ treatment on the Ti-terminated TiN (111) surface is more likely to make the TiN surface become an N-terminated TiN (111) surface, which results in making a lot of N-terminated TiN (111) surfaces, having a very reactive nature for B_2_H_6_ bond dissociation. As a result, the effect of N_2_ treatment serves as a catalyst to decompose B_2_H_6_. From the deep understanding of the effect of H_2_ and N_2_ during the B_2_H_6_ dosing process, the use of proper gas treatment is required for the improvement of the W nucleation layers.

## Abstract 2

Our results showed the effects of H_2_ and N_2_ treatment on TiN surfaces, using density functional theory calculations. These imply that the understanding of gas treatment gives us insight into improving the W ALD process for future memory devices.

## Graphical abstract

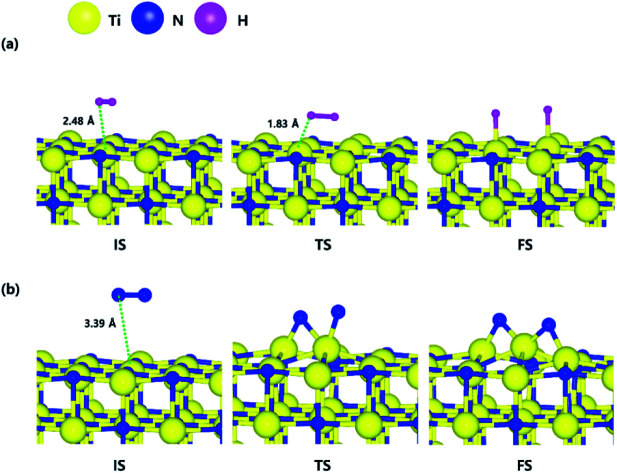

## 1. Introduction

As the demand for miniaturized and highly integrated devices in the electronics industry increases, conformal film deposition techniques that can precisely control thickness at the atomic scale are becoming very important.^1,2^ Nitride materials, such as titanium nitride, silicon nitride, have been deposited using conventional deposition systems such as low-pressure chemical vapor deposition (LPCVD)^3,4^ and plasma-enhanced chemical vapor deposition (PECVD).^5,6^ Another deposition technique, atomic layer deposition (ALD),^7–9^ is the most prevalent technique for the fabrication of new memory devices due to the excellent step coverage and high conformality on extremely high aspect ratio structures. The ALD processes utilize well-controlled sequential surface reactions to achieve uniform and conformal films.^10,11^

As one of the indispensable materials in the fabrication of future memory devices, tungsten (W) has been used in the metal gate deposition process due to the capability of depositing lower resistive films than other candidate materials, which results in the enhancement of the device performance.^12,13^ In the fabrication of memory devices, tungsten films have most widely been deposited using the ALD process by alternatively exposing W precursors such as tungsten hexafluoride (WF_6_) and reducing agents such as diborane (B_2_H_6_) in an ABAB… sequence. In the ALD W processes, the B_2_H_6_ dosing process plays a critical role in the deposition of W films with low resistivity and in the removal of residual fluorine (F) atoms on the surface.^14–16^ However, since the aspect ratio increases as the size of the memory device becomes smaller, it becomes difficult to deposit a thin film having excellent step coverage and conformality due to the problem of a seam or void being formed in the process of filling the W metal gate. This problem is a major obstacle to the development of future memory devices.^17,18^ To tackle this problem, the theoretical comprehension of the ALD W process is required due to the experimentally limited observations on the sub-nanometer scale. During the ALD W process, H_2_ and N_2_ molecules are used as both a purging gas at the purge time and a dilute gas (5% B_2_H_6_ + 95% H_2_ or N_2_) at the B_2_H_6_ dosing time.^15^ Although a few experimental results on ALD W have been reported, there has been no theoretical report on the effect of H_2_ and N_2_ treatment used in the ALD W process.

In our previous study, we reported that the severe problems, such as seams or voids, in filling the W metal gate for memory devices could be attributed to the difference in the deposition rate of the W film depending on the orientations of the TiN surfaces by analyzing the dissociation reaction of B_2_H_6_ on three different TiN surfaces using the density functional theory (DFT)^19^ calculation method. Since this previous study does not give information on the H_2_ and N_2_ treatment for the B_2_H_6_ dosing process, we want to report how important the use of proper gas treatment could be for B_2_H_6_ bond dissociation.

Previous *ab initio* molecular dynamics (AIMD) simulation results have shown that the presence of N vacancies catalyzes N_2_ dissociative chemisorption on the TiN (001) surface. N_2_ dissociation is never observed at the defect-free TiN (001) surface.^20^ The phenomena were also observed for the vanadium nitride (001) surface.^21^ D. G. Sangiovanni *et al.* demonstrated that the Ti and N adatom diffusion mechanism on TiN (001) involves direct hopping onto a lower layer and push-out/exchange by classical molecular dynamics (CMD) and AIMD simulations at temperatures ranging from 1200 to 2400 K.^22–25^ They also carried out Ti and N adatom migration pathways on the TiN (111) surface and showed that Nad species are considerably more mobile than Tiad on TiN (111), contrary to their previous results on TiN (001) by CMD based on the modified embedded atom method (MEAM) at temperatures ranging from 600 to 1800 K.^26^ A similar study was also carried out by Yuan Ren *et al.*, showing that the diffusion energy of the Ti adatom is greater than that of the N adatom, confirmed by first principles calculations.^27^ C. Tholander *et al.* reported that Ti, Al, and N adatom mobilities on TiN (001), (011) and (111) surfaces, in general, are fastest on TiN (001), slower on (111), slowest on (011).^28^

In this study, we present a first-principles study based on DFT calculations to investigate the effects of H_2_ and N_2_ treatment on TiN surfaces for the B_2_H_6_ dosing process. In the first step, the decomposition processes of H_2_ and N_2_ treatment on the TiN surface were carefully analyzed. Then, in the second step, the decomposition processes of B_2_H_6_ molecules on the H_2_- and N_2_-treated TiN surfaces were analyzed to determine H_2_ and N_2_ treatment effects, respectively. From our calculated results in this study, the structure of the TiN surfaces can be changed as the TiN surface is exposed to large amounts of H_2_ or N_2_ molecules during the B_2_H_6_ dosing process. As a result, the changed structure of the TiN surfaces can have a significant impact on the ALD W process because the underlying surfaces can have significant effects on the characteristics of the subsequent W nucleation layers.^29,30^ The TiN surfaces have been widely utilized as a glue/barrier layer for subsequent W nucleation.^31^ Three different planes of TiN surfaces, TiN (001), Ti-terminated TiN (111), and N-terminated TiN (111) were taken into account because poly-crystalline TiN layers with (001) and (111) preferred orientations were mainly observed in the deposition of TiN films.^32,33^ The dissociative reaction pathways and reaction energetics of both H_2_ and N_2_ on three different TiN surfaces were investigated to explore the effect of H_2_ and N_2_ treatment for the B_2_H_6_ dosing process. It is expected that the comparative analysis of both H_2_ and N_2_ would give us insight into how important the use of proper gas treatment could be for improving the quality of the subsequent W layer during the W ALD process. This study will help to understand how H_2_ and N_2_ treatment plays an important role in the B_2_H_6_ dissociation reaction and ultimately provide new important information for improving the W ALD process.

## 2. Computational methods

In our theoretical results, all DFT calculations were performed using the Vienna *ab initio* simulation package (VASP) program with the Perdew–Burke–Ernzerhof (PBE) functional in the generalized gradient approximation (GGA).^34,35^ We used the PBE-D2 functional with a correction to the conventional Kohn–Sham DFT energy to treat the vdW interactions for all TiN surface calculations.^36^ The projector augmented wave (PAW) method was used to describe the interaction between valence electrons and ion cores.^37^ TiN (001) and TiN (111) surfaces with the B1-NaCl structure were used as the reactive surfaces with the B_2_H_6_ precursor. The optimized lattice parameter of TiN was *a*_0_ = 4.259 Å, which overestimated, somewhat, the experimental value (*a*_0_ = 4.24 Å)^38^ since PBE functionals generally tend to overestimate the lattice parameters. Compared to our PBE based calculated lattice parameter, previous research papers for DFT calculations of TiN reported that an optimized lattice parameter of TiN using the PBE functional is close to 4.254 Å.^25,39^ Another related paper using the Armiento Mattsson (AM05) approximation reported yields of 4.220 Å.^40^ For the TiN (001) surface, a 4-layer slab of (2 × 2) supercell with 64 atoms was considered. For comparison, the TiN surfaces with Ti-terminated and N-terminated (111) orientations were considered with a 5-layer slab of (2 × 2) supercell with 45 atoms.

For all TiN surfaces, such as TiN (001), Ti-terminated TiN (111), and N-terminated TiN (111), vacuum gaps with values of 23.7 Å, 25.4 Å, and 25.6 Å, respectively, in the *z*-direction were included to avoid interactions between adjacent slabs. Valence orbitals were described by a plane-wave basis set with the cutoff energy of 400 eV. Electronic energies were calculated with a self-consistent-field (SCF) tolerance of 10^−4^ eV on the total energy. Ultrasoft Vanderbilt-type pseudopotentials^41^ were used to describe the interactions between ions and electrons. A 3 × 3 × 3 Monkhorst *k*-point mesh for bulk TiN was chosen. The Brillouin zone for three different TiN surfaces was sampled with a 3 × 3 × 1 Monkhorst–Pack *k*-point mesh. Geometry optimization was performed by minimizing the forces of all atoms to less than 0.02 eV Å^−1^ with the total energy of the system converged to within 10^−4^ eV during self-consistent iterations. In addition, we calculated total energies for various configurations to determine the energy barrier for the dissociative adsorption of H_2_ and N_2_ on the TiN surfaces in the first step, and for one of B_2_H_6_ on H-covered TiN surfaces in the second step.

To optimize adsorption structures, we considered two orientations and three positions of H_2_ and N_2_ on the three different TiN surfaces. The details of all six cases are shown in the ESI (Fig. S1–S3†). The optimized adsorption structures with the lowest energies (ESI, Tables S1–S3†) were used in this paper. We also checked three orientations and three positions of B_2_H_6_ on both H-covered Ti-terminated TiN (111) and H-covered N-terminated TiN (111) surfaces. The details of all nine cases are shown in the ESI (Fig. S4 and S5†). The optimized adsorption structures with the lowest energies (ESI, Tables S4 and S5†) were used in this paper. To find the optimized reaction path for the B_2_H_6_ bond dissociation on the H-covered TiN (111) surface, we considered three reaction paths, namely, path a, path b, and path c in the ESI (Fig. S8 and S9†). The optimized reaction path (path a) with the lowest overall reaction energy (ESI, Tables S8 and S9†) was used in this paper. To check the differences between PBE-D2 based calculations for convergence criteria of forces, such as 20 meV Å^−1^, and 1 meV Å^−1^, we carried out DFT calculations of H_2_ and N_2_ dissociative reactions on three TiN surfaces in the ESI (Table S10†). In those calculations, energy profiles with very similar results were obtained for two cases, *i.e.*, PBE-D2 (<20 meV Å^−1^), PBE-D2 (<1 meV Å^−1^). To be more specific, the difference in activation energies between the two cases is very small (maximum difference: 0.007 eV, minimum difference: 0). Moreover, the difference in reaction energies between the two cases was not found. We believe it is reasonable to use the criteria of forces less than 20 meV Å^−1^.

It is worth noting that PBE-D2 generally results in a well-known and physically understood estimation of the dissociative reactions of molecules on various surfaces. Various computational approaches can be utilized to correct the shortcomings of approximate DFT calculations, including GW corrections^42^ or some exact Hartree–Fock (HF) exchange in the modern hybrid density functional (B3LYP, PBE0, HSE, *etc.*),^43–45^ which can lead to substantially improved band gaps; however, they are significantly computationally demanding. Since all our DFT calculations are addressed to bond dissociative reactions of various molecules, we believe it is reasonable and quantitative to investigate the dissociative reactions of various molecules on TiN surfaces using the PBE-D2 theory. In addition, PBE-D2 predictions have proven useful for prediction of the dissociative reactions of various molecules on different surfaces as shown by the numerous studies on the dissociation of different large molecules on Au (gold),^46^ oxygen reduction reaction on Co(acetylaetonate)_2_,^47^ water dissociation on mackinawite (FeS),^48,49^ and the dissociative reaction of silicon precursor on Si.^50^

To calculate the transition state, the distance between the two dissociative atoms was slightly separated, and energy relaxation was performed with the constrained distance. The same procedures were carried out until the force between two dissociative atoms became almost zero at the saddle point energy. This procedure for the calculation of the transition state is required to find not only the accurate final state but also transition state, especially in complicated systems such as B_2_H_6_ dissociation. After this procedure, we used the nudged elastic band method^51^ using the calculated final state to check the accurate transition state. During surface relaxation in our DFT calculations, no obvious surface reconstruction was found in different surfaces, such as TiN (001), Ti-terminated TiN (111), N-terminated TiN (111), which has been confirmed in another report.^52^

## 3. Results and discussion

### 3.1. H_2_ and N_2_ dissociative chemisorption on TiN (001)

**Fig. 1:**
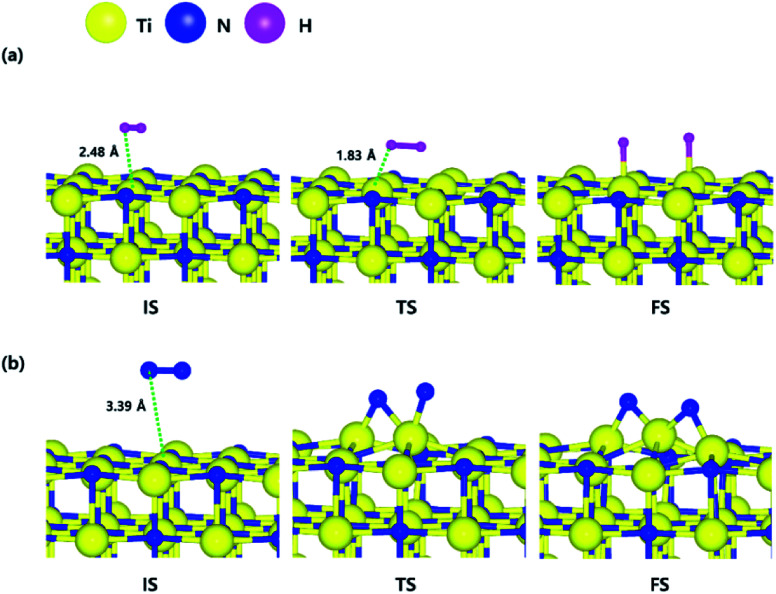
The optimized initial, transition, and final structures of the dissociative chemisorption step for (a) H_2_ and (b) N_2_ on the TiN (001) surface.

The optimized structures of the initial, transition and final states for the H_2_ and N_2_ dissociative chemisorption step on the TiN (001) surface are shown in Fig. 1(a) and (b). The calculated energy diagram of H_2_ and N_2_ decomposition on the three different TiN surfaces is shown in Fig. 4. The initial state (IS) in Fig. 1(a) presents the optimized structure with the lowest adsorption energy of H_2_ on the surface. The final state (FS) in Fig. 1(a) shows that dissociated H atoms from H_2_ molecule react with titanium atoms of the TiN (001) surface after H–H bond dissociation because the binding energy on titanium sites of the TiN (001) surface is 0.42 eV larger compared to the nitrogen sites in our calculated results.

The reaction energy can be calculated as the energy difference between the initial state and the final state. As shown in Fig. 4, the calculated reaction energy of H_2_ on the TiN (001) surface is 1.04 eV, which shows that the reaction is endothermic and energetically unfavorable. The activation energy from the initial state to the final state is 1.68 eV with the transition state as shown in Fig. 4. The N_2_ dissociative chemisorption step on the TiN (001) surface is shown in Fig. 1(b). It was found that the reaction energy is 3.25 eV (Fig. 4), which indicates that the reaction is thermodynamically endothermic and unfavorable. The activation energy from the initial state to the final state is 4.92 eV with the transition state as shown in Fig. 4.

### 3.2. H_2_ and N_2_ dissociative chemisorption on Ti-terminated TiN (111)

**Fig. 2:**
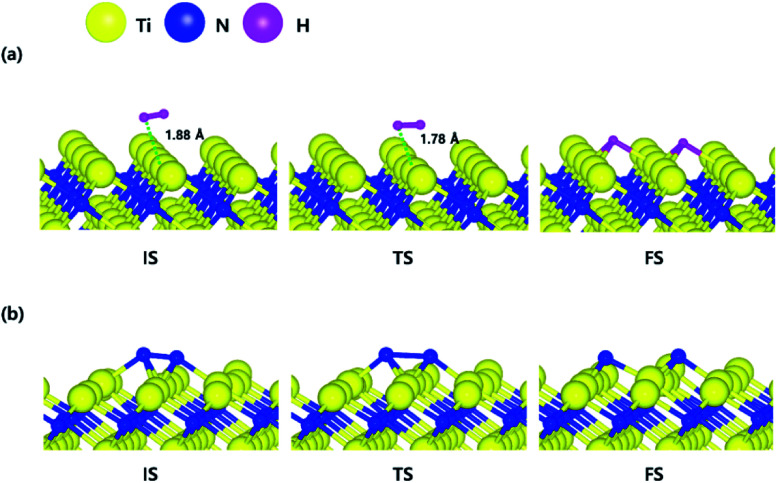
The optimized initial, transition, and final structures of the dissociative chemisorption step for (a) H_2_ and (b) N_2_ on the Ti-terminated TiN (111) surface.

The decomposition mechanism of H_2_ and N_2_ was also studied on the Ti-terminated TiN (111) surface to estimate the difference between TiN (001) and TiN (111) surfaces. The optimized initial, transition, and final structures of H_2_ and N_2_ on the Ti-terminated TiN (111) surface are shown in Fig. 2. It was found that dissociated H atoms and N atoms in the final state were adsorbed on the hollow site made by three Ti atoms (Site number 3 in Fig. S2†). As shown in Fig. 2(a) and 4, it was found that the reaction energy of H_2_ is −2.10 eV, which indicates that the reaction is exothermic and energetically favorable. The activation energy from the initial state to the final state is 0.11 eV with the transition state as shown in Fig. 4, indicating that this reaction has a small energy barrier. As shown in Fig. 2(b) and 4, the adsorption energy of N_2_ is −3.44 eV, showing that the adsorption is energetically favorable. The reaction energy of N_2_ is −2.06 eV in Fig. 4, which indicates that the reaction is exothermic. The activation energy from the initial state to the final state is 0.92 eV with the transition state as shown in Fig. 4. The energy diagram for the H_2_ and N_2_ decomposition on Ti-terminated TiN (111) differs from the TiN (001) as illustrated in Fig. 4. It demonstrates that both H–H and N–N bond dissociation steps on the Ti-terminated TiN (111) surfaces are more facile than the TiN (001) surface due to the smaller activation energies of dissociation on the Ti-terminated TiN (111) surface. Moreover, the reactions of both H_2_ and N_2_ are energetically favorable, with their reaction energies of −2.10 eV, −2.06 eV, respectively. The high reactivity of both molecules on the Ti-terminated TiN (111) surface is most likely to be because the surface has triple dangling bonds per atom, which make the surface even more reactive than the TiN (001) surface. To be more specific, the number of dangling bonds on the Ti-terminated TiN (111) surface is more than that of the TiN (001), so that the bond dissociation of both H_2_ and N_2_ is more favorable on the former. This analysis was confirmed by the higher adsorption of both H and N atoms on the Ti- terminated (111) surface compared to the TiN (001) surface as shown in Table 1. Due to the aforementioned reasons, this surface can also reduce the energy barriers of the H_2_ and N_2_ decomposition as compared to the TiN (001) surface.

**Table 1:** Binding energies (eV) of both H and N atoms on the most stable site of TiN (001), Ti-terminated TiN (111) and N-terminated TiN (111) surfaces

Surface	H	N
TiN (001)	2.93	5.79
Ti-terminated TiN (111)	4.61	11.0
N-terminated TiN (111)	5.73	Not bound

### 3.3. H_2_ and N_2_ dissociative chemisorption on N-terminated TiN (111)

**Fig. 3:**
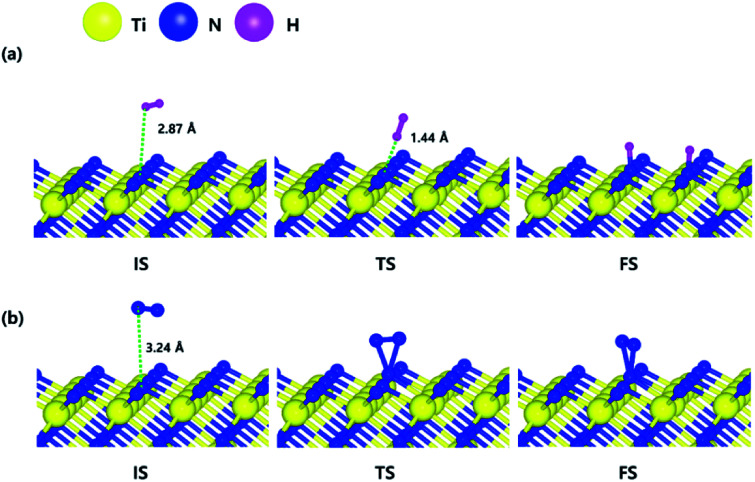
The optimized initial, transition, and final structures of the dissociative chemisorption step for (a) H_2_ and (b) N_2_ on the N-terminated TiN (111) surface.

**Fig. 4:**
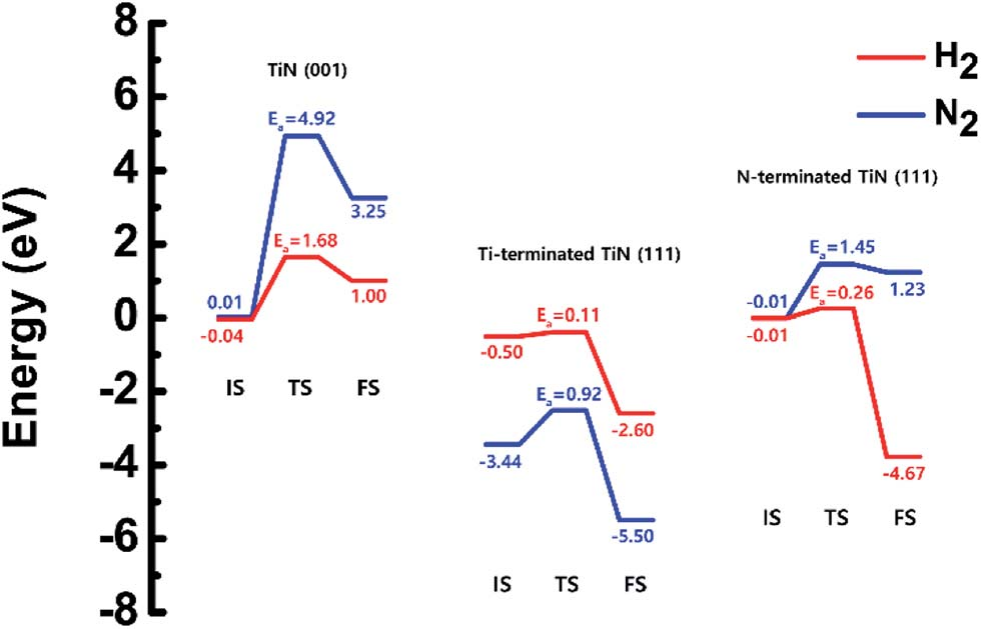
Calculated energy diagram of H_2_ and N_2_ decomposition on the TiN (001), the Ti-terminated TiN (111), and N-terminated TiN (111) surface.

The optimized initial, transition, and final structures of H_2_ and N_2_ on the N-terminated TiN (111) surface are displayed in Fig. 3. It was found that dissociated H atoms in the final state were adsorbed on the hollow site made by three N atoms (Site number 3 in Fig. S3†). As shown in Fig. 3(a) and 4, the reaction energy of H_2_ is −4.66 eV, which indicates that the reaction is exothermic. The activation energy from the initial state to the final state is 0.26 eV with the transition state, indicating that this reaction requires a low energy barrier. However, as for the N_2_ in Fig. 3(b) and 4, the reaction energy of N_2_ is 1.24 eV, which indicates that the reaction is endothermic. In this case, N_2_ molecules were not dissociated on the surface. The activation energy from the initial state to the final state is 1.45 eV with the transition state. We found that the H–H bond breaking on the N-terminated TiN (111) surfaces was much more facile compared to those of both TiN (001) and Ti-terminated TiN (111) surfaces, as shown in Fig. 4. This result is primarily because the binding energy (*E*_b_ = 5.73 eV) of H atoms on the N-terminated TiN (111) surface is the highest among the three different TiN surfaces, as shown in Table 1. Furthermore, the decomposition of H_2_ on the N-terminated TiN (111) surface is energetically favorable due to the downhill reaction and small energy barrier for H–H bond breaking. However, the N-terminated TiN (111) surface was not advantageous for breaking the N–N bond because N_2_ was not dissociated on the N-terminated TiN (111) surface. Although the N_2_ molecule has a larger bond dissociation energy (*E*_dissosciation_ = 9.45 eV)^53^ than H_2_ (*E*_dissosciation_ = 4.36 eV)^53^ due to the triple bonding nature of N_2_, this N_2_ molecule could be dissociated on the Ti-terminated TiN (111) surface. We suggest that this phenomenon is attributed to a large binding energy (*E*_b_ = 11.0 eV) of the N atom on the Ti-terminated TiN (111) surface, as depicted in Table 1.

### 3.4. The effect of N_2_ treatment on TiN surfaces during the ALD process

As mentioned in Section 3.1, it is difficult to decompose both H_2_ and N_2_ on the TiN (001) surface because of the reactions in Fig. 4 are energetically unfavorable. However, since Ti-terminated TiN (111) can decompose H_2_ and N_2_ as shown Fig. 4, H-covered Ti-terminated TiN (111) and N-terminated TiN (111) surfaces can be made. In the case of N terminated TiN (111), the N–N bond dissociation is not possible, but H–H bond dissociation is possible, so the H-covered N-terminated TiN (111) surface can be made. Fig. 2(b) and 4 show that N_2_ treatment on the Ti-terminated TiN (111) surface is more likely to make the surface an N-terminated TiN (111) surface, which results in making a lot of N-terminated TiN (111) surfaces under the ALD process. Our previous study^19^ has shown that the N-terminated TiN (111) surface among the three TiN surfaces is the best surface for the B_2_H_6_ decomposition reaction. The combination of our previous study^19^ and this study provides new information on the effect of N_2_ treatment, which plays the role of a catalyst to decompose B_2_H_6_. Since both the results from our previous study and this study explain the effect of N_2_ treatment well enough, we have focused on the investigation of the H_2_ effect in this study. The decomposition processes of B_2_H_6_ on both the H covered Ti-terminated TiN (111) and the H-covered N-terminated TiN (111) surface were analyzed in detail to investigate the effect of H_2_ treatment. For the TiN (001) surface, the H-covered TiN (001) surface is excluded from the text because it is energetically unstable and H atoms on the surface are desorbed as H_2_.

### 3.5. B_2_H_6_ dissociative chemisorption on H-covered Ti-terminated TiN (111)

**Fig. 5:**
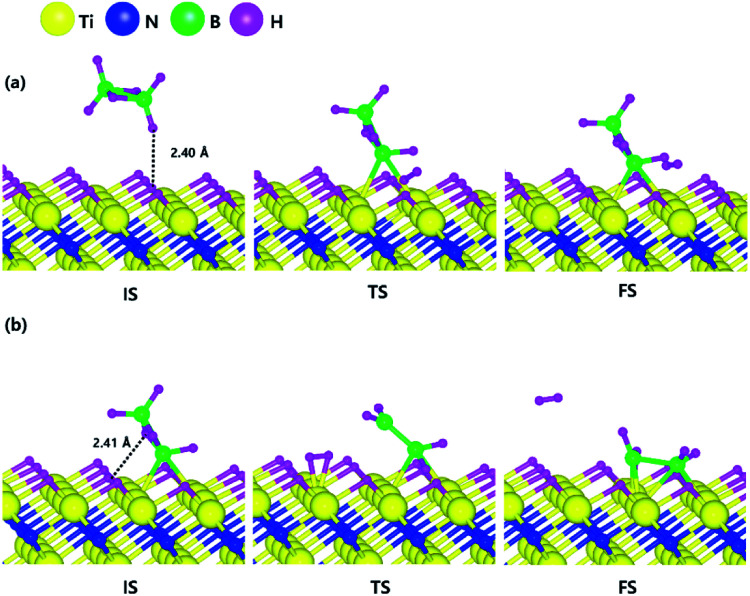
Dissociative chemisorption of B_2_H_6_ on the H-covered Ti-terminated TiN (111) surface: (a) the first reaction step (b) the second reaction step.

**Fig. 6:**
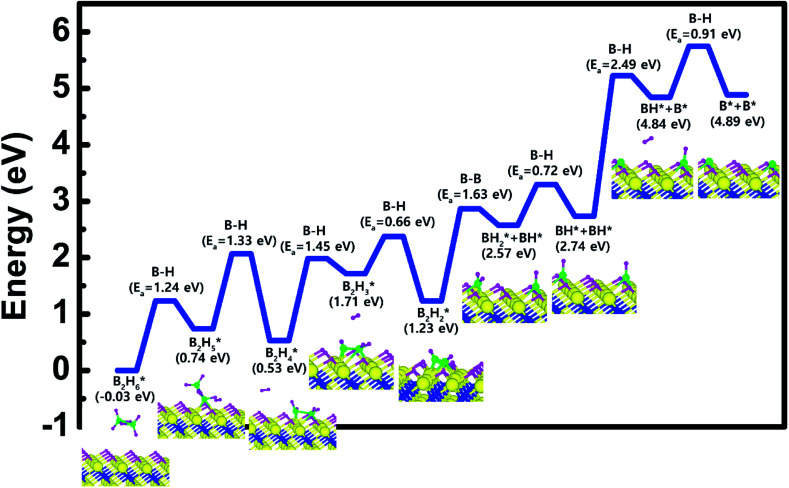
Calculated energy diagram of B_2_H_6_ decomposition on the H-covered Ti-terminated TiN (111) surface.

Based on the results shown in Section 3.2, when the Ti-terminated TiN (111) surface is subjected to H_2_ treatment, the H_2_ molecule can be easily decomposed because of the low energy barrier for H_2_ bond dissociation on the surface, and thus the H-covered Ti-terminated TiN (111) surface can be made. Fig. 5 shows the decomposition reaction mechanism of B_2_H_6_ molecules when the H-covered Ti-terminated TiN (111) surface is formed after H_2_ treatment. Fig. 5(a) shows the first reaction step where the H atoms of the B_2_H_6_ molecules adsorbed on the TiN surface react with the H atoms on the TiN surface, desorbing H_2_, and the remaining B_2_H_5_ is bound to the surface. It was found that the dissociated B_2_H_5_ molecule is adsorbed on the hollow site made by three Ti atoms (Site number 4 in Fig. S4†). The reaction energy is 0.77 eV in Fig. 6, indicating that the reaction is endothermic. The activation energy is 1.24 eV with the transition state in Fig. 6. Fig. 5(b) shows the second reaction step where the H atoms of the B_2_H_5_ molecules adsorbed on the surface react with the H atom of the TiN surface then desorb into H_2_. As for the second reaction step, it shows that the reaction energy is −0.11 eV with an activation energy of 1.33 eV in Fig. 6.

To complete the overall reaction energetics of B_2_H_6_ for the optimized reaction path, the calculated energy diagram of B_2_H_6_ decomposition on the H-covered Ti-terminated TiN (111) surface is displayed in Fig. 6. The detailed structures of B_2_H_6_ during the overall reaction pathway on the surface for transition state calculations can be found in the ESI (Fig. S6†). During the reaction of the B_2_H_6_ precursor, this calculation shows that the overall reaction process is endothermic, with a calculated overall reaction energy of 4.92 eV in Table 2. This result indicates that the B_2_H_6_ dissociative chemisorption on H-covered Ti-terminated TiN (111) is energetically unfavorable due to the uphill reactions and high activation energies that range from a minimum of 0.66 eV to a maximum of 2.49 eV in Table 2. This implies that the low reactivity of B_2_H_6_ with the surface is due to the presence of the H-covered surface, compared to our previous results,^19^ as shown in Table 2, reporting that the dissociative reaction of B_2_H_6_ is energetically favorable on Ti-terminated TiN (111) surface. As a result, an effect of the H_2_ treatment on the surface is to passivate the TiN surface to prevent it from reacting with the B_2_H_6_ molecule.

**Table 2:** Comparison of the minimum and maximum activation energies (*E*_a,minimum_, *E*_a,maximum_, eV) and overall reaction energies (*E*_rxn,overall_, eV) of B_2_H_6_ bond dissociation on the H-covered TiN and the TiN surfaces

Surface	Bond dissociation	*E*_a,minimum_ (eV)	*E*_a,maximum_ (eV)	*E*_rxn,overall_ (eV)	Note
H-covered Ti-terminated TiN (111)	B–B & B–H	0.66	2.49	4.92	This study
H-covered N-terminated TiN (111)	B–B & B–H	0.40	1.65	1.26	This study
Ti-terminated TiN (111)	B–B & B–H	0.07	0.93	−0.88	Our previous study^19^
N-terminated TiN (111)	B–B & B–H	Barrier-less	0.39	−19.0	Our previous study^19^

### 3.6. B_2_H_6_ dissociative chemisorption on H-covered N-terminated TiN (111)

**Fig. 7:**
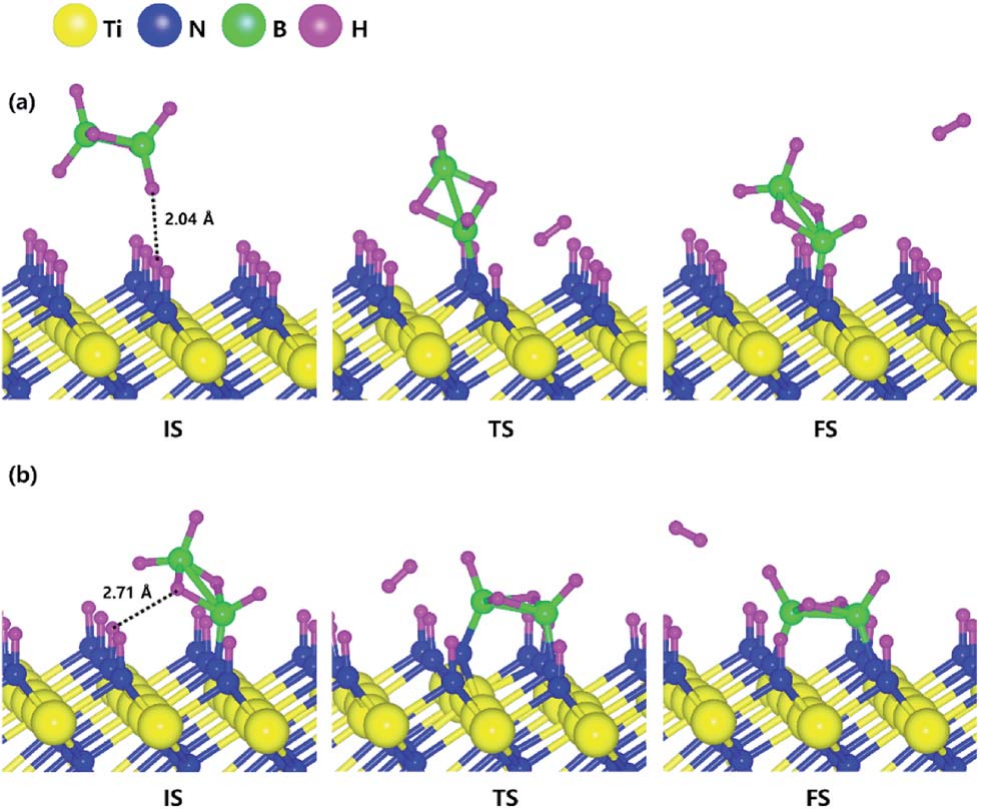
Dissociative chemisorption of B_2_H_6_ on the H-covered Ti-terminated TiN (111) surface: (a) the first reaction step (b) the second reaction step.

**Fig. 8:**
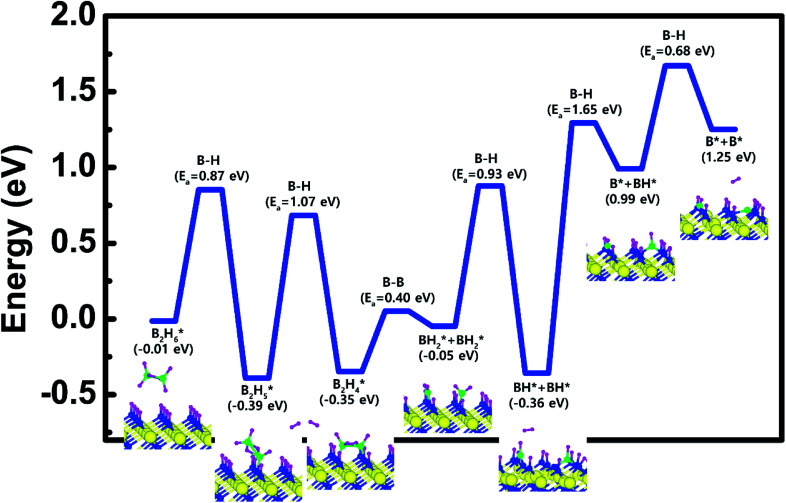
Calculated energy diagram of B_2_H_6_ decomposition on the H-covered N-terminated TiN (111) surface.

Based on the results shown in Section 3.3, when the N-terminated TiN (111) surface is subjected to H_2_ treatment, the H_2_ molecule can be easily dissociated because of the low barrier for the H_2_ bond dissociation on the surface, and thus the H-covered N-terminated TiN (111) surface can be made. Fig. 7 shows the decomposition reaction mechanism of B_2_H_6_ molecules when the H-covered N-terminated TiN (111) surface is formed after H_2_ treatment. Fig. 7(a) shows the first reaction step where the H atoms of the B_2_H_6_ molecule adsorbed on the TiN surface react with the H atoms on the TiN surface, desorbing H_2_, and the remaining B_2_H_5_ binds to the surface. The reaction energy is −0.38 eV in Fig. 8, indicating that the reaction is exothermic. The activation energy is 0.87 eV with the transition state in Fig. 8. Fig. 7(b) shows the second reaction step where the H atom of the B_2_H_5_ molecule adsorbed on the surface reacts with the H atom of the TiN surface to be desorbed into H_2_. As for the second reaction step, it shows that the reaction energy is 0.04 eV with an activation energy of 1.07 eV in Fig. 8.

The entire energy diagram for the B_2_H_6_ decomposition on the H-covered N-terminated TiN (111) is illustrated in Fig. 8. The detailed structures of B_2_H_6_ during the overall reaction pathway on the surface for transition state calculations can be found in the ESI (Fig. S7†). During the reaction of the B_2_H_6_ precursor, this calculation shows that the overall reaction process is endothermic with a calculated overall reaction energy of 1.26 eV in Table 2. Although the overall reaction from the 1^st^ reaction step to the 7^th^ reaction step (ESI, Fig. S7†) is energetically unfavorable, B_2_H_6_ dissociative reactions can occur from the 1^st^ reaction step to the 5^th^ reaction step because the overall reaction energy from the 1^st^ to 5^th^ step is −0.35 eV as shown in Fig. 8, meaning that the reaction is energetically favorable. From the 5^th^ reaction step, two remaining BH species are difficult to dissociate due to the uphill reactions of B–H bond breaking with energy barriers of 1.65 eV, and 0.68 eV, respectively in Fig. 8.

Unlike the H-covered Ti-terminated TiN (111), the H-covered N-terminated TiN (111) surface can dissociate the B_2_H_6_ molecule into BH species up to 5^th^ reaction step. We suggest that those remaining BH species and H atoms would be desorbed as BF_3_ and HF from WF_6_ in the next ALD cycle.

However, compared to the N-terminated TiN (111) in our previous results,^19^ the dissociative reaction of B_2_H_6_ is much more favorable on the N-terminated TiN (111) surface than the H-covered N-terminated TiN (111) surface as shown in Table 2. This implies that the low reactivity of B_2_H_6_ with the surface is attributed to the presence of an H-covered surface. As a result, an effect of the H_2_ treatment on the surface is to passivate the TiN surface to prevent it from reacting with the B_2_H_6_ molecules.

## 4. Discussion

Table 2 shows the minimum and maximum activation energies, and overall reaction energies required for B_2_H_6_ bond dissociation on the H-covered TiN and the TiN surfaces. The information on the TiN (001) surface is excluded because it is difficult to make an H-covered surface as mentioned above in Section 3.4. In addition, it was reported in our previous study that the B_2_H_6_ dissociative reaction on the TiN (001) surface is energetically unfavorable,^19^ meaning that the TiN (001) is not suitable for the B_2_H_6_ dosing process.

As shown in Table 2, B_2_H_6_ dissociative reactions on both the H-covered Ti-terminated TiN (111) surface and the H-covered N-terminated TiN (111) surface require large overall reaction energies and activation energies, meaning that those reactions are energetically unfavorable. However, B_2_H_6_ dissociative reactions on both the Ti-terminated TiN (111) surface and the N-terminated TiN (111) surface are exothermic, meaning that those reactions are energetically favorable. In conclusion, H_2_ treatment on both Ti-terminated TiN (111) surface and N-terminated TiN (111) surface converts these surfaces into H-covered surfaces, leading to the degradation of the B_2_H_6_ dissociative reactions. As a result, H_2_ treatment has an effect of passivating the TiN surfaces. However, an effect of N_2_ treatment on the TiN surface is more likely to make the surface an N-terminated TiN (111) surface under the ALD process as mentioned above in Section 3.4, which leads to a lot of N-terminated TiN (111) surfaces. Table 2 shows that the B_2_H_6_ dissociative reaction on the N-terminated TiN (111) surface is much more energetically favorable than the Ti-terminated TiN (111) surface because it has a much lower reaction energy. As a result, N_2_ treatment has the effect of making the TiN surfaces more reactive for B_2_H_6_ bond dissociation. In the next ALD cycle after the B_2_H_6_ dosing process, the WF_6_ molecule is generally used for W deposition. Since boron (B) adatoms on the TiN surface would react with the F atoms of WF_6_, the BF_3_ desorption process would occur on the surface and therefore, a uniform W film could be deposited.

Our results imply that making a lot of N-terminated TiN (111) surfaces, by N_2_ treatment, plays an important role in improving the properties of the subsequent W nucleation layers during the W ALD process because easily dissociated B adatoms on the surface could dissociate the WF_6_ molecule and desorb into BF_3_. Since W nucleation layers are experimentally difficult to grow using only WF_6_ molecules without the B_2_H_6_ dosing process,^54^ B_2_H_6_ should be easily dissociated on the TiN surfaces for obtaining high-quality W nucleation layers during the W ALD process. Although H_2_ molecules play a role in lowering the reactivity of B_2_H_6_ on the TiN surfaces, this molecule can be useful to remove residual F atoms, which degrade the quality of the W layer. It has been reported that ALD W deposition under H_2_ exposure helps to remove residual F atoms by the desorption of HF.^55^ The previous experimental results and our theoretical results provide insight into how to design the ALD W deposition process to develop the W films for highly integrated devices.

## 5. Conclusions

In summary, we investigated the effects of H_2_ and N_2_ treatment on TiN surfaces for the B_2_H_6_ dosing process based on DFT calculation. Since the H_2_ molecule is easily dissociated on both Ti-terminated TiN (111) and N-terminated TiN (111) surfaces, H-covered Ti-terminated TiN (111) and H-covered N-terminated TiN (111) surfaces can be made. In our DFT calculated results, H_2_ treatment on the TiN surfaces convert the surfaces into H-covered TiN surfaces, which results in lowering the reactivity of the B_2_H_6_ precursor since the overall reactions of the B_2_H_6_ on the H-covered TiN surfaces are energetically less favorable than the TiN surfaces. As a result, an effect of the H_2_ treatment is to decrease the reactivity of the B_2_H_6_ molecule on the TiN surface. However, N_2_ treatment on the Ti-terminated TiN (111) surface is more likely to make the surface an N-terminated TiN (111) surface, which results in making a lot of N-terminated TiN (111) surfaces with very reactive nature for B_2_H_6_ bond dissociation. As a result, the effect of N_2_ treatment serves as a catalyst to decompose B_2_H_6_. Although the N_2_ molecule is more useful for B_2_H_6_ bond dissociation than H_2_ in regard to the reactivity, the H_2_ molecule has a greater advantage for the removal of residual F atoms than N_2_. From the understanding of the effect of H_2_ and N_2_ during the B_2_H_6_ dosing process, the use of proper gas treatment is required for the improvement of the W nucleation layers. These results imply that the deep understanding of the role of H_2_ and N_2_ treatment will provide insight for improving the W ALD process for future memory devices.

## Conflicts of interest

There are no conflicts to declare.

## Supplementary Material

RA-008-C8RA02622J-s001
