# Supplementary material for: Effects of H2 and N2 treatment for B2H6 dosing process on TiN surfaces during atomic layer deposition: an ab initio study
Source: RSC Adv. 2018 Jun 8;8(38):21164–73. doi: 10.1039/c8ra02622j (PMC9080878; doi:10.1039/c8ra02622j)
Supplement: RA-008-C8RA02622J-s001 [file RA-008-C8RA02622J-s001.pdf]

Electronic Supplementary Material (ESI) for RSC Advances.
This journal is © The Royal Society of Chemistry 2018

Electronic Supplementary Information

Effects of H_2 and N_2 treatment for B_2H_6 dosing process on TiN surfaces during atomic layer deposition: An ab-initio study

Hwanyeol Park,[a] Sungwoo Lee,[a] Ho Jun Kim,[b] Daekwang Woo,[c] Se Jun Park,[c] Kangsoo Kim,[c] Euijoon Yoon,*[,a, d] Gun-Do Lee*[,a, d]

[a]Department of Materials Science and Engineering, Seoul National University, Seoul 08826, Korea.

[b]Department of Mechanical Engineering, Dong-A University, Busan 49315, South Korea.

[c]Memory Thin Film Technology Team, Giheung Hwaseong Complex, Samsung Electronics, 445-701, South Korea.

[d]Research Institute of Advanced Materials and Inter-University Semiconductor Research Center, Seoul National University, Seoul 08826, South Korea.

Corresponding authors: eyoon@snu.ac.kr; gdlee@snu.ac.kr

This supporting information explains the detailed structures of adsorption, transition, and reaction state of B_2H_6 on both H-covered Ti-terminated TiN and H-covered N-terminated TiN surfaces. The adsorption energy (E_{ads}) was calculated using

$E_{ads} = E_{tot,ads} - (E_{surf} + E_{pre})$

where $E_{tot,ads}$, E_{surf}, and E_{pre} are the total energy of the system after adsorption, and the energy of the surface only and the energy of the precursor only, respectively. The activation energy (E_a) was calculated using

$E_a = E_{tot,tran} - E_{tot,b.tr}$

where $E_{tot,tran}$ and $E_{tot,ads}$ are the total energy of the transition state and the total energy before transition, respectively. The reaction energy (E_{rxn}) was calculated using

$E_{rxn} = E_{tot,a.tr} - E_{tot,b.tr}$

where $E_{tot,a.tr}$, and $E_{tot,b.tr}$ are the total energies of the system after transition and after transition, respectively.

We considered two orientations and three positions of H_2 and N_2 above the TiN (001), Ti-terminated TiN (111), and N-terminated (111) TiN surfaces as shown in Figures S1-S3. As for the two orientations, first one is that those molecules were vertical to the surface. Second one is that those molecules were horizontal to the surface. Also, three different positions were considered on the surface:

For TiN (001) surface, (1) Ti site, (2) Hollow site, (3) N site

For Ti-terminated TiN (111) surface, (1) Ti site (2) N site, (3) Hollow site

For N-terminated TiN (111) surface, (1) N site (2) Ti site, (3) Hollow site

The adsorption energies of H_2 and N_2 calculated on three different TiN surfaces for each orientation and position were summarized in Table S1-S3.

In case of geometry 2 in Figures S1-S3, adsorption positions were selected when one molecule atom is adsorbed on the specific position since almost initial structures with geometry 2 after structural relaxation become final structures that one molecule atom is tilted to the adsorption position. Since final structures for both <001> and <110> directions after structural relaxation have almost similar results for both molecular

orientations on the same position of TiN and adsorption energies, we chose the word “geometry 2” rather than <100> or <110> directions to avoid the use of duplicated values for adsorption energies.

We also checked three orientations and three positions of B_2H_6 above the H-covered Ti-terminated TiN (111), and H-covered N-terminated (111) TiN surfaces as shown in Figures S4-S5. As for the three orientations, first one is that the B-B bond of the precursor was vertical to the surface. Second one is that the B-B bond of one was horizontal to the surface with two hydrogen atoms facing towards the surface, and third one is that the B-B bond of one was horizontal to the surface with only one hydrogen is facing towards the surface. Also, three different positions were considered on the surface:

For H-covered Ti-terminated TiN (111) surface, (1) H site (2) Ti site, (3) N site

For H-covered N-terminated TiN (111) surface, (1) H site (2) Ti site, (3) Hollow site

The adsorption energies of B_2H_6 precursor calculated on two different TiN surfaces for each orientation and position were summarized in Table S4-S5.

Figures S6-S7 show the optimized structures at specific reaction states, such as initial, transition, and final step, for B_2H_6 decomposition on the H-covered TiN surfaces. Both initial and final states were calculated at the position of remaining B, H, and BH_x species on the most stable site of those surfaces. Table S6-S7 indicate the activation energies and reaction energies for the overall reactions of B_2H_6 decomposition on the H-covered TiN surfaces. These tables provide information on whether the B_2H_6 reaction is energetically stable or unstable for each reaction step.

Figure S8-S9 show the calculated overall energy diagrams of B_2H_6 decomposition for path a, b, and c on the H-covered TiN (111) surface. There are three reaction paths. As shown in Figure S6, path a is the path where the H atom of the B_xH_y molecule adsorbed on the surface react with the H atom of the TiN surface to be desorbed into H_2 during 1st, 2nd, 3rd, and 7th reaction steps. At 5th reaction step, B-B bond breaking from B_2H_2 occurs. For 4th, 6th, and 8th reaction steps, H atom of B_xH_y molecule is adsorbed on the site where H atom of the surface is empty due to H_2 desorption in previous reaction steps of 3rd, 5th, and 7th, respectively. Path b is the path where the H atom of the B_xH_y molecule adsorbed on the surface react with the H atom of the TiN surface to be desorbed into H_2 during all reaction steps without the 7th reaction step for B-B bond breaking. Path c is where the H atom of the B_xH_y molecule adsorbed on the surface react with the H atom of the TiN surface to be desorbed into H_2 during 1st, 2nd, 3rd, and 6th reaction steps. For 4th and 6th reaction steps, H atom of B_xH_y molecule is adsorbed on the site where H atom of the surface is empty due to H_2 desorption in previous reaction steps of 3rd and 5th, respectively. At the 7th reaction step, B-B bond breaking from B_2 occurs. Table S8-S9 indicate comparison of minimum and maximum activation energies (E_a, minimum, E_a, maximum, eV) and overall reaction energies (E_{rxn}, overall, eV) of B_2H_6 dissociation for path a, b, and c on the H-covered TiN surfaces.

Table S10 shows the comparison of activation energies (E_a, eV) and reaction energies (E_{rxn}, eV) of both H_2 and N_2 dissociation for three TiN surfaces under two different cases, such as PBE-D2 (<20 meV/Å), and PBE-D2 (<1 meV/Å). This calculation was performed to check the differences between PBE-D2 based calculations for convergence criteria of forces, such as, 20 meV/Å, and 1 meV/Å.

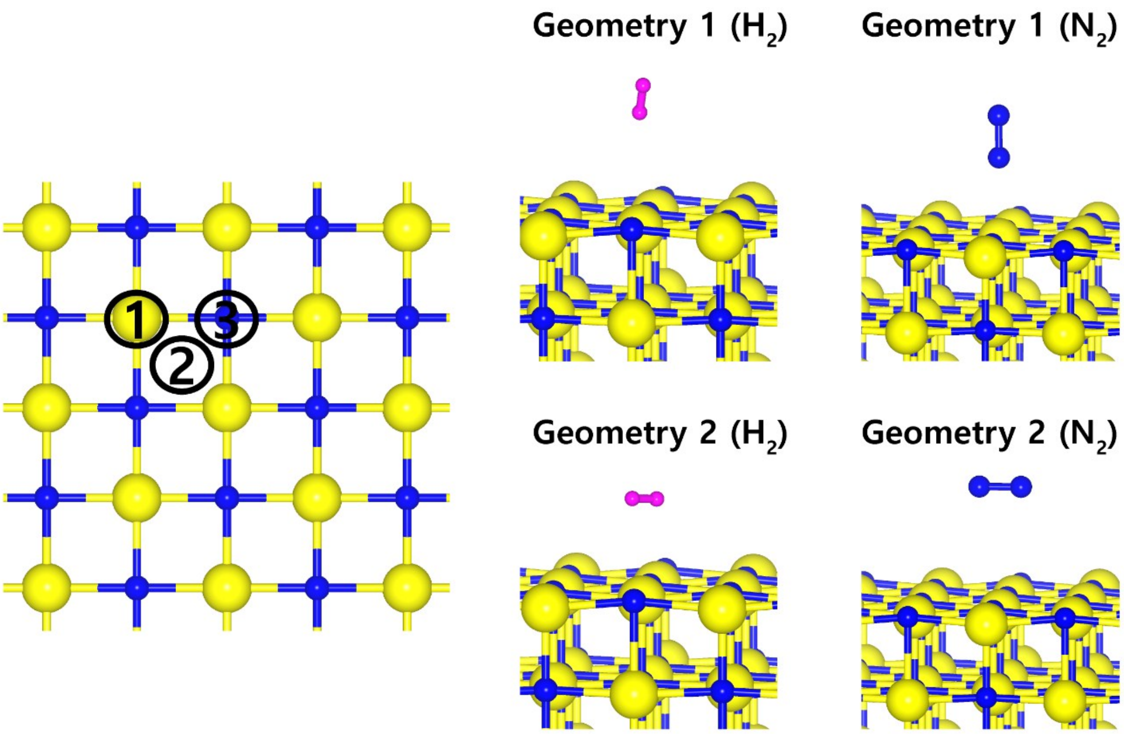

Figure S1. Two orientations and three positions of H_2 and N_2 on the TiN (001) surface. The yellow, blue, and pink spheres represent Ti, N, and H atoms, respectively.

Table S1. The adsorption energies of H_2 and N_2 calculated on the TiN (001) surface for each orientation and position.

(a) H_2

Geometry	Position	E ads (meV)
1	1	-6.59
1	2	-7.56
1	3	-19.94
**2**	**1**	**-37.94**
2	2	13.69
2	3	23.29

(b) N_2

Geometry	Position	E ads (meV)
1	1	22.44
1	2	47.77
1	3	-3.93
**2**	**1**	**-10.56**
2	2	1389.76
2	3	79.5

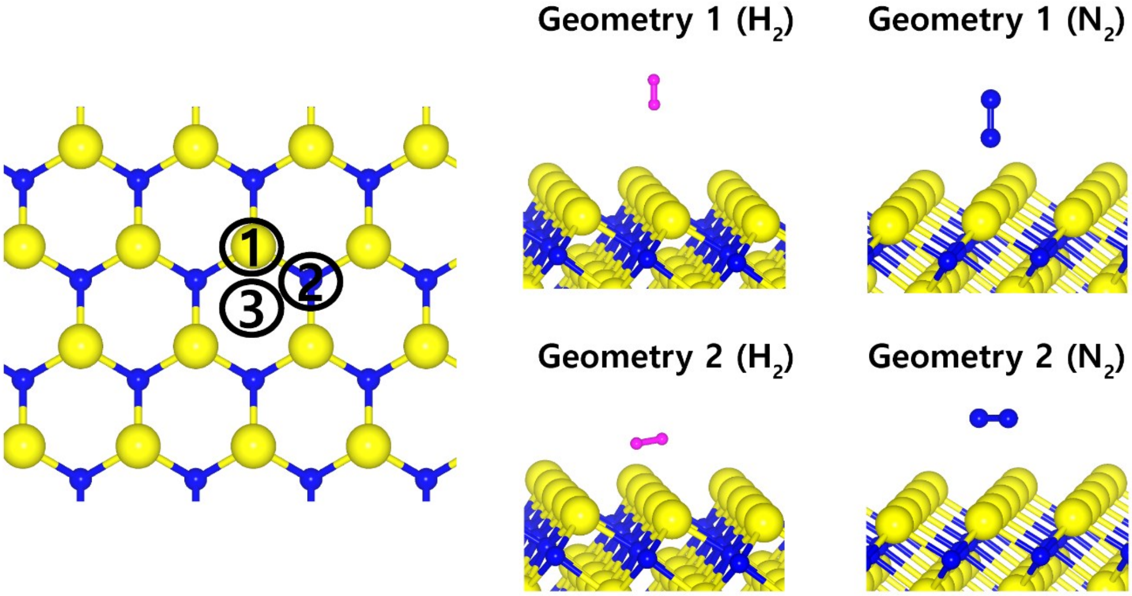

Figure S2. Two orientations and three positions of H_2 and N_2 on the Ti-terminated TiN (111) surface.

Table S2. The adsorption energies of H_2 and N_2 calculated on the Ti-terminated TiN (111) surface for each orientation and position.

(a) H_2

Geometry	Position	E ads (meV)
1	1	14.99
1	2	13.01
1	3	11.92
**2**	**1**	**-498.74**
2	2	101.02
2	3	101.05

(b) N_2

Geometry	Position	E ads (meV)
1	1	-790.89
1	2	78.86
1	3	129.47
**2**	**1**	**-3437.43**
2	2	-3144.97
2	3	207.32

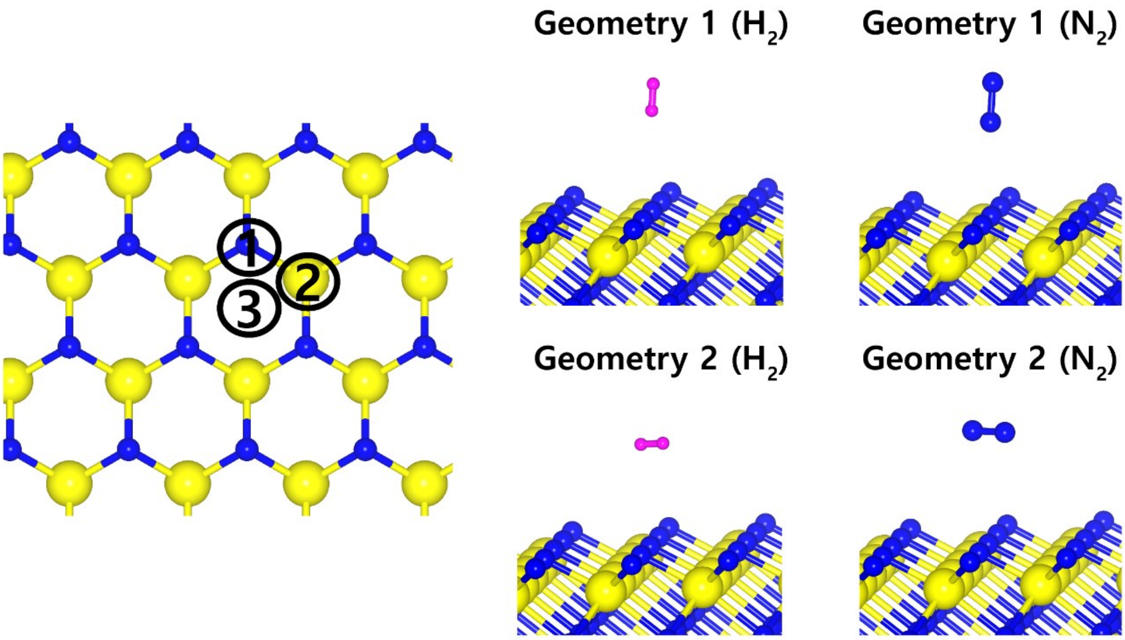

Figure S3. Two orientations and three positions of H_2 and N_2 on the N-terminated TiN (111) surface.

Table S3. The adsorption energies of H_2 and N_2 calculated on the N-terminated TiN (111) surface for each orientation and position.

(a) H_2

Geometry	Position	E ads (meV)
1	1	-21.72
1	2	-19.21
1	3	-11.25
**2**	**1**	**-31.82**
2	2	-17.48
2	3	-20.29

(b) N_2

Geometry	Position	E ads (meV)
1	1	95.41
1	2	13.78
1	3	13.78
**2**	**1**	**-11.63**
2	2	17.95
2	3	12.39

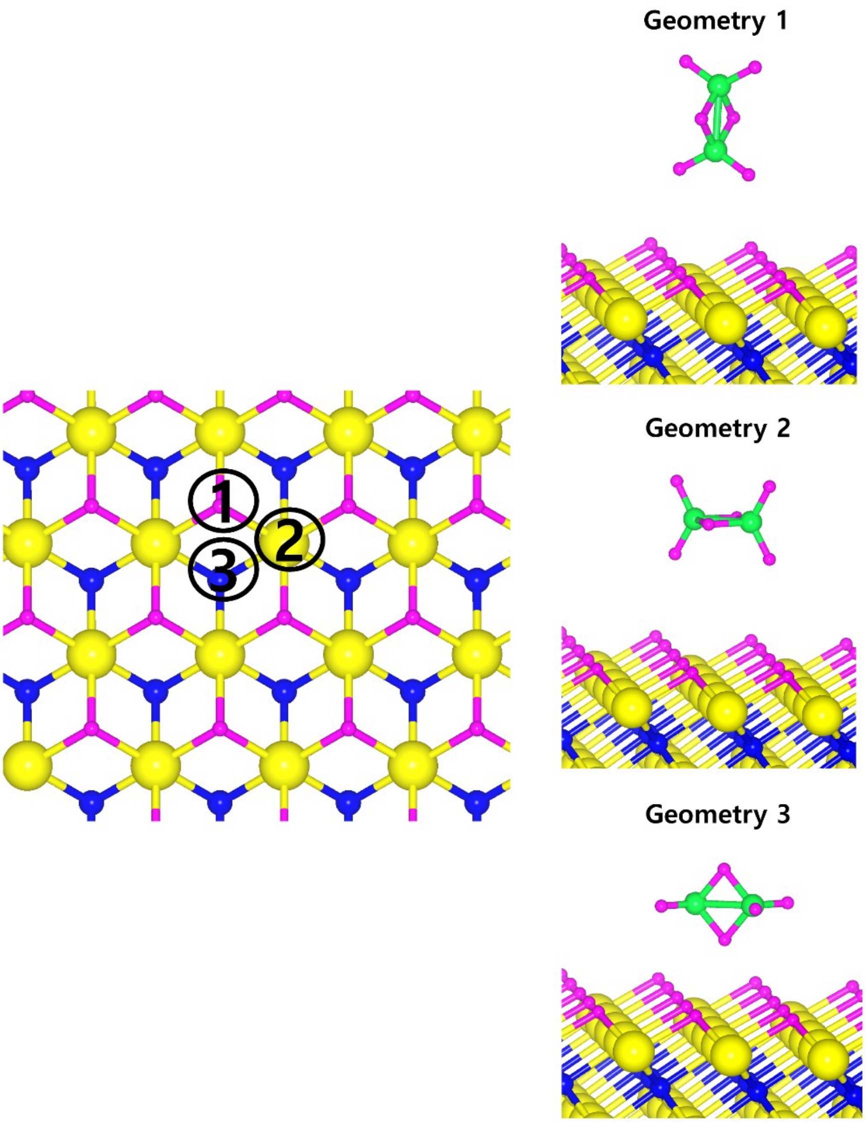

Figure S4. Three orientations and three positions of B_2H_6 on the H-covered Ti-terminated TiN (111) surface.

Table S4. The adsorption energies of B_2H_6 calculated on the H-covered Ti-terminated TiN (111) surface for each orientation and position.

Geometry	Position	E ads (meV)
1	1	1.57
1	2	37.51
1	3	62.63
**2**	**1**	**-26.76**
2	2	-1.59
2	3	-22.21
3	1	94.97
3	2	45.1
3	3	-4.22

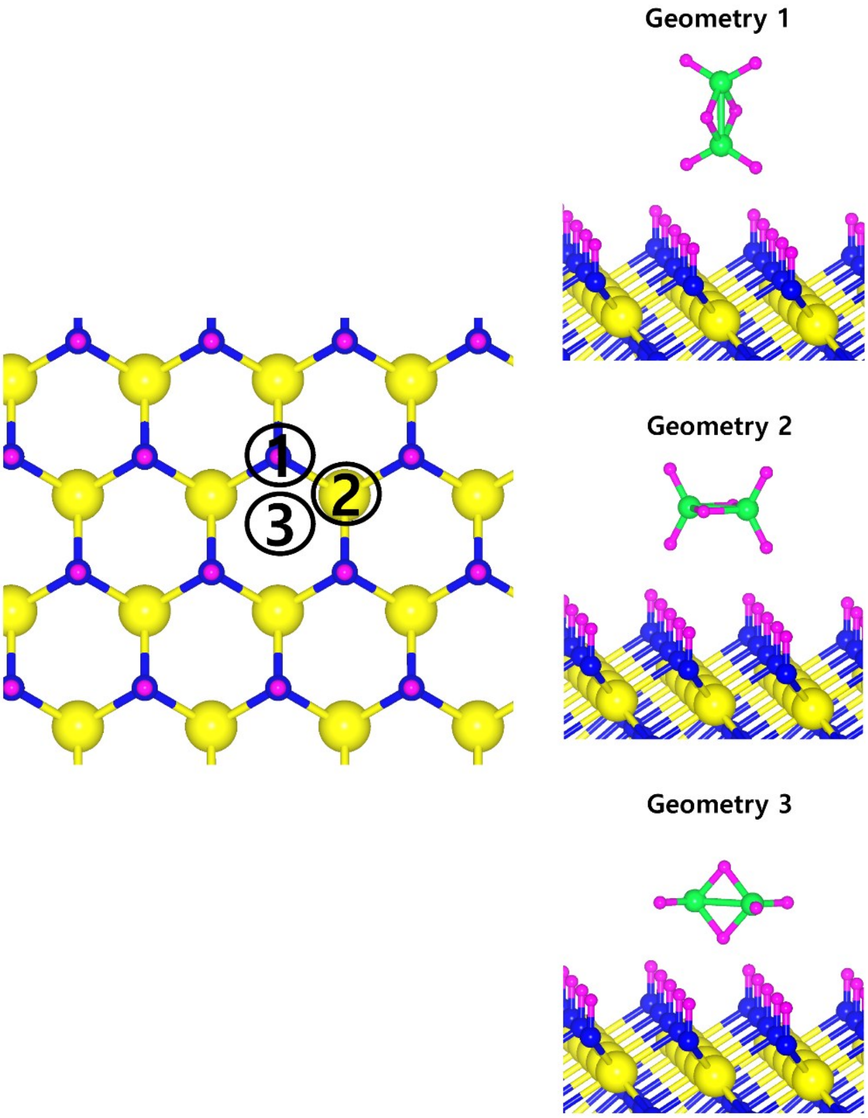

Figure S5. Three orientations and three positions of B_2H_6 on the H-covered N-terminated TiN (111) surface.

Table S5. The adsorption energies of B_2H_6 calculated on the H-covered N-terminated TiN (111) surface for each orientation and position.

Geometry	Position	E ads (meV)
1	1	34.87
1	2	43.27
1	3	44.13
**2**	**1**	**-13.20**
2	2	-8.40
2	3	-7.19
3	1	40.13
3	2	10.13
3	3	9.21

3rd reaction step : B-H bond dissociation

IS TS FS

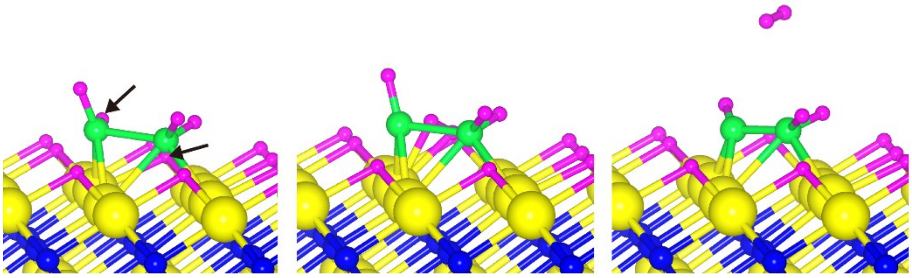

4th reaction step : B-H bond dissociation

IS TS FS

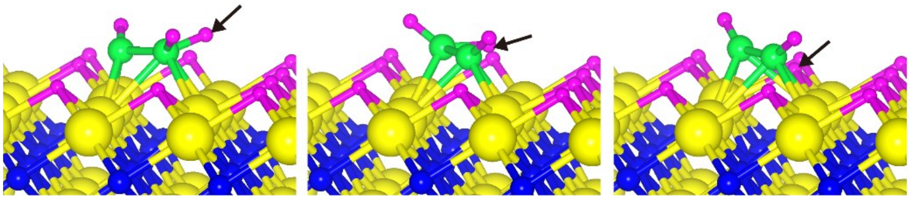

5th reaction step : B-B bond dissociation

IS TS FS

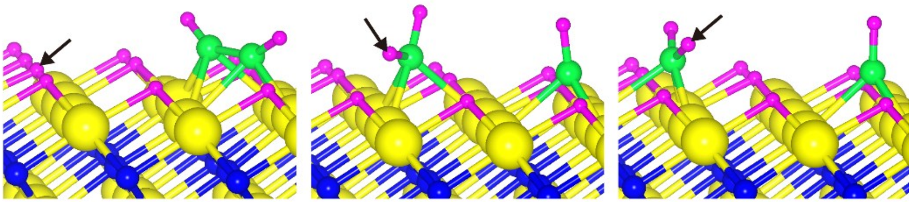

6th reaction step : B-H bond dissociation

IS TS FS

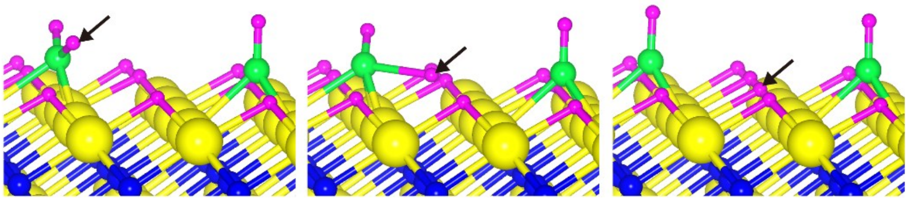

7th reaction step : B-H bond dissociation

IS TS FS

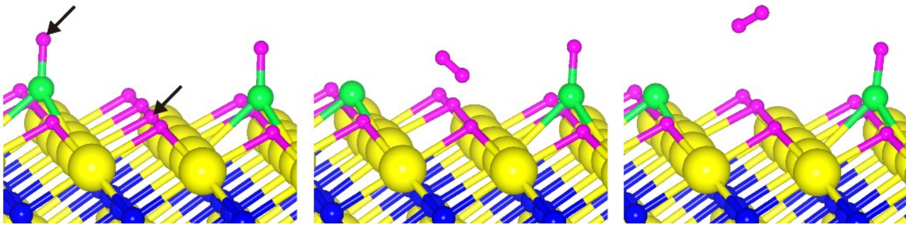

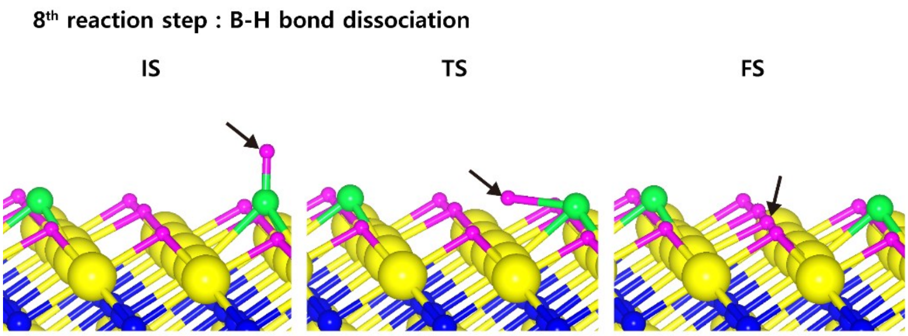

Figure S6. Initial (IS), transition (TS), and final (FS) states of intermediate reactions from the 3rd reaction step to the 7th reaction step on the H-covered Ti-terminated TiN (111) surface.

Table S6. Activation energies (E_a, eV) and reaction energies (E_{rxn}, eV) of B_2H_6 dissociation on the H-covered Ti-terminated TiN (111) surface.

Reacton step	Bond dissociation	E_a (eV)	E_{rxn} (eV)
step 1	B-H	1.24	0.77
step 2	B-H	1.33	-0.21
step 3	B-H	1.45	1.18
step 4	B-H	0.66	-0.48
step 5	B-H	1.63	1.34
step 6	B-B	0.72	0.17
step 7	B-H	2.49	2.10
step 8	B-H	0.91	0.05

3rd reaction step : B-B bond dissociation

IS TS FS

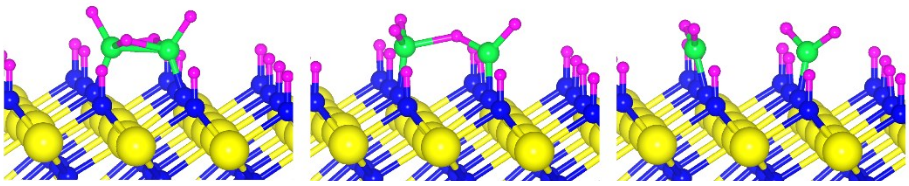

4th reaction step : B-H bond dissociation

IS TS FS

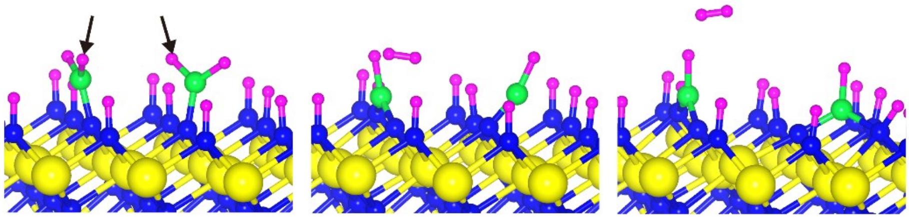

5th reaction step : B-H bond dissociation

IS TS FS

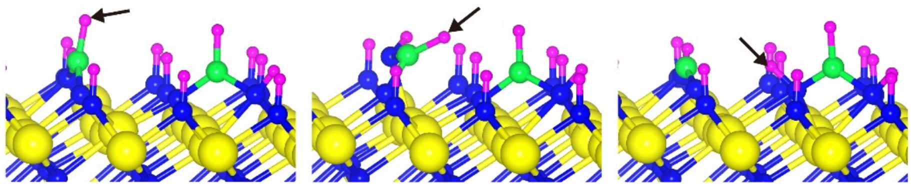

6th reaction step : B-H bond dissociation

IS TS FS

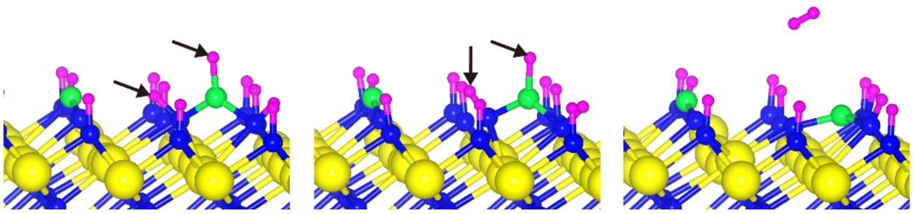

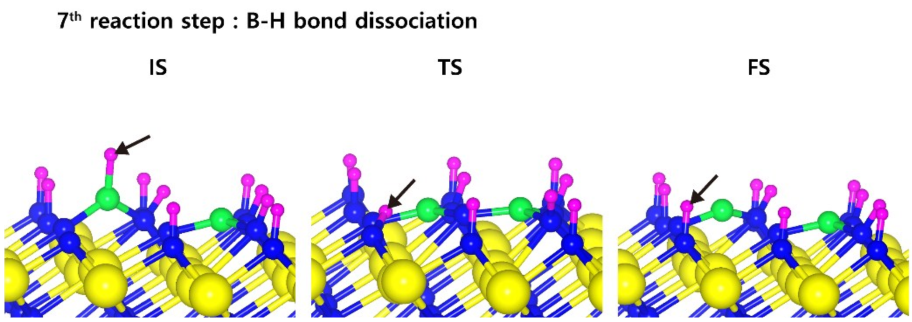

Figure S7. Initial (IS), transition (TS), and final (FS) states of intermediate reactions from the 3rd reaction step to the 7th reaction step on the H-covered N-terminated TiN (111) surface.

Table S7. Activation energies (E_a, eV) and reaction energies (E_{rxn}, eV) of B_2H_6 dissociation on the H-covered N-terminated TiN (111) surface.

Reacton step	Bond dissociation	E_a (eV)	E_{rxn} (eV)
step 1	B-H	0.87	-0.38
step 2	B-H	1.07	0.04
step 3	B-B	0.40	0.30
step 4	B-H	0.93	-0.31
step 5	B-H	1.65	1.35
step 6	B-H	0.68	0.26

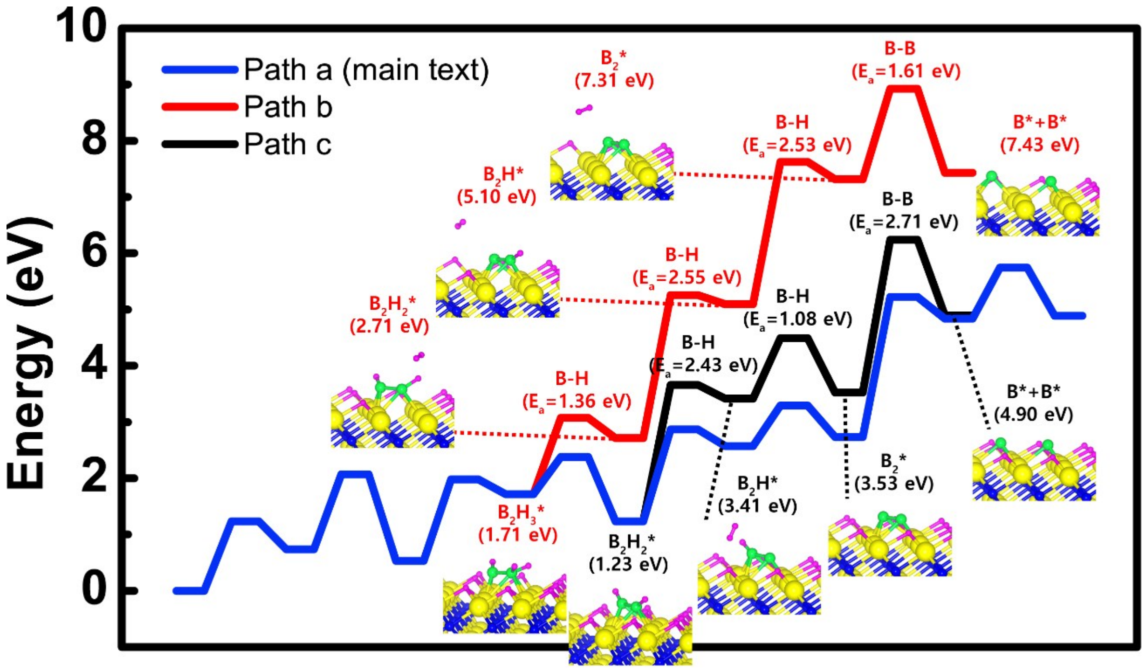

Figure S8. Calculated overall energy diagram of B_2H_6 decomposition for path a, b, and c on the H-covered Ti-terminated TiN (111) surface.

Table S8. Comparison of minimum and maximum activation energies (E_a, minimum, E_a, maximum, eV) and overall reaction energies (E_{rxn}, overall, eV) of B_2H_6 dissociation for path a, b, and c on the H-covered Ti-terminated TiN surfaces.

Surface	Bond dissociation	$E_{a,\ minimum}$ (eV)	$E_{a,\ maximum}$ (eV)	$E_{rxn,\ overall}$ (eV)	Note
H-covered Ti-terminated TiN (111)	B-B & B-H	0.66	2.49	4.92	Path a (main text)
		1.36	2.55	7.46	Path b
		1.08	2.71	4.93	Path c

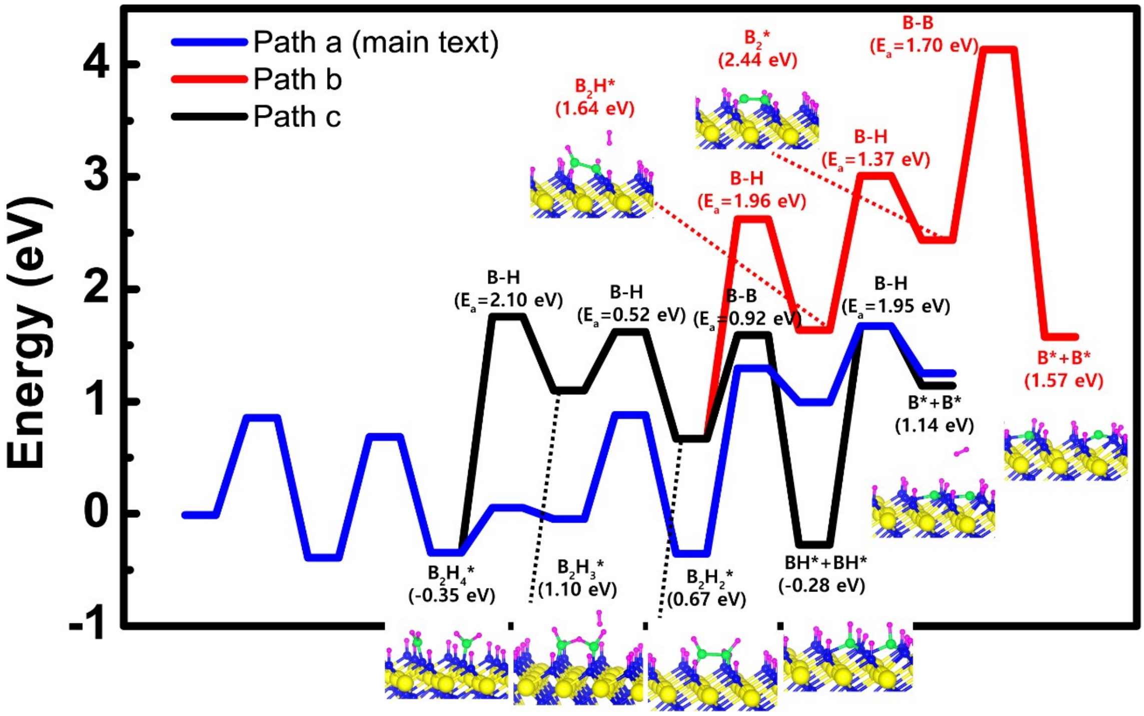

Figure S9. Calculated overall energy diagram of B_2H_6 decomposition for path a, b, and c on the H-covered N-terminated TiN (111) surface.

Table S9. Comparison of minimum and maximum activation energies (E_a, minimum, E_a, maximum, eV) and overall reaction energies (E_{rxn}, overall, eV) of B_2H_6 dissociation for path a, b, and c on the H-covered N-terminated TiN surfaces.

Surface	Bond dissociation	$E_{a,\ minimum}$ (eV)	$E_{a,\ maximum}$ (eV)	$E_{rxn,\ overall}$ (eV)	Note
H-covered N-terminated TiN (111)	B-B & B-H	0.40	1.65	1.26	Path a (main text)
		0.52	2.10	1.58	Path b
		0.52	2.10	1.15	Path c

Table S10. Comparison of activation energies (E_a, eV) and reaction energies (E_{rxn}, eV) of both H_2 and N_2 dissociation for three TiN surfaces under two different cases, such as PBE-D2 (<20 meV/Å), and PBE-D2 (<1 meV/Å).

Surface	Molecule	PBE-D2 (<20meV/Å)		PBE-D2 (<1meV/Å)	
		E_a (eV)	E_{rxn} (eV)	E_a (eV)	E_{rxn} (eV)
TiN (001)	H_2	1.677	1.035	1.675	1.035
	N_2	4.924	3.242	4.917	3.242
Ti-terminated TiN (111)	H_2	0.110	-2.097	0.110	-2.097
	N_2	0.921	-2.064	0.921	-2.064
N-terminated TiN (111)	H_2	0.257	-4.401	0.256	-4.401
	N_2	1.449	1.246	1.449	1.246
